# Inflammation in hepatocellular carcinoma progression: a “Single Wick, dual interwoven strands” regulatory framework

**DOI:** 10.3389/fimmu.2026.1822039

**Published:** 2026-04-22

**Authors:** Chao Wu, Jing Dong, Shangru Sui, Xiuyan Tan, Na Zhu

**Affiliations:** 1 Department of General Surgery, Qingdao Central Hospital, University of Health and Rehabilitation Sciences, Qingdao, Shandong, China; 2 Department of Clinical Trial Research Center, Qingdao Central Hospital, University of Health and Rehabilitation Sciences, Qingdao, Shandong, China; 3 Department of Pharmacy, Qingdao Central Hospital, University of Health and Rehabilitation Sciences, Qingdao, Shandong, China

**Keywords:** chronic inflammation, hepatocellular carcinoma, inflammatory cells, inflammatory cytokines, tumor microenvironment

## Abstract

Hepatocellular carcinoma (HCC) is one of the most prevalent and lethal malignancies worldwide, with chronic liver inflammation as the core driver of its initiation and progression. Regardless of etiology, HCC development follows the classic progressive pathway of “chronic inflammation - liver fibrosis - cirrhosis - liver cancer”. In this review, we propose an innovative “Single Wick, Dual Interwoven Strands” regulatory framework to systematically delineate the role of inflammation in the entire continuum of HCC progression. We define chronic liver inflammation as the “Wick” – the core driver that initiates and sustains the pro-tumor cascade, while inflammatory cells and inflammatory cytokines act as the two interwoven functional “Strands” that execute the pro-carcinogenic effects of inflammation via a tightly regulated interactive network. Their synergistic crosstalk drives five core terminal biological outcomes of HCC progression: invasion and metastasis, angiogenesis, immune escape, metabolic reprogramming, and therapeutic resistance. Based on this framework, we dissect the core mechanisms of inflammation-driven HCC, summarize the latest advances in inflammation-targeted diagnostic, therapeutic and preventive strategies, and highlight the clinical translational prospects of this framework. This work provides systematic theoretical guidance for early prevention, precise treatment and prognosis improvement of HCC.

## 1 Introduction

Primary liver cancer is the sixth most common malignant tumor worldwide and the third leading cause of cancer-related death, of which hepatocellular carcinoma (HCC) accounts for more than 90% (1). In recent years, despite continuous innovations in treatment modalities such as targeted therapy and immune checkpoint inhibitors, the 5-year overall survival rate of HCC patients remains less than 20%. The core reasons for this are the complex pathogenesis of HCC, low early diagnosis rate, high postoperative recurrence rate, and the fact that most patients are already at an advanced stage at the time of diagnosis (2). Numerous clinical and basic studies have confirmed that chronic liver inflammation is the “initiator” and “accelerator” of HCC occurrence and development, with more than 90% of HCC cases arising from a background of chronic liver inflammation (3). Regardless of etiology, including HBV/HCV infection, MASH, and alcohol-associated liver disease, liver injury triggers a sustained inflammatory cascade that drives the classic “chronic inflammation - liver fibrosis - cirrhosis - HCC” progression pathway (2–4). During this process, persistent inflammatory stimulation drives sequential tissue damage and repair, establishing a pro-tumorigenic microenvironment that predisposes hepatocytes to malignant transformation (5–8).

**Figure 1:**
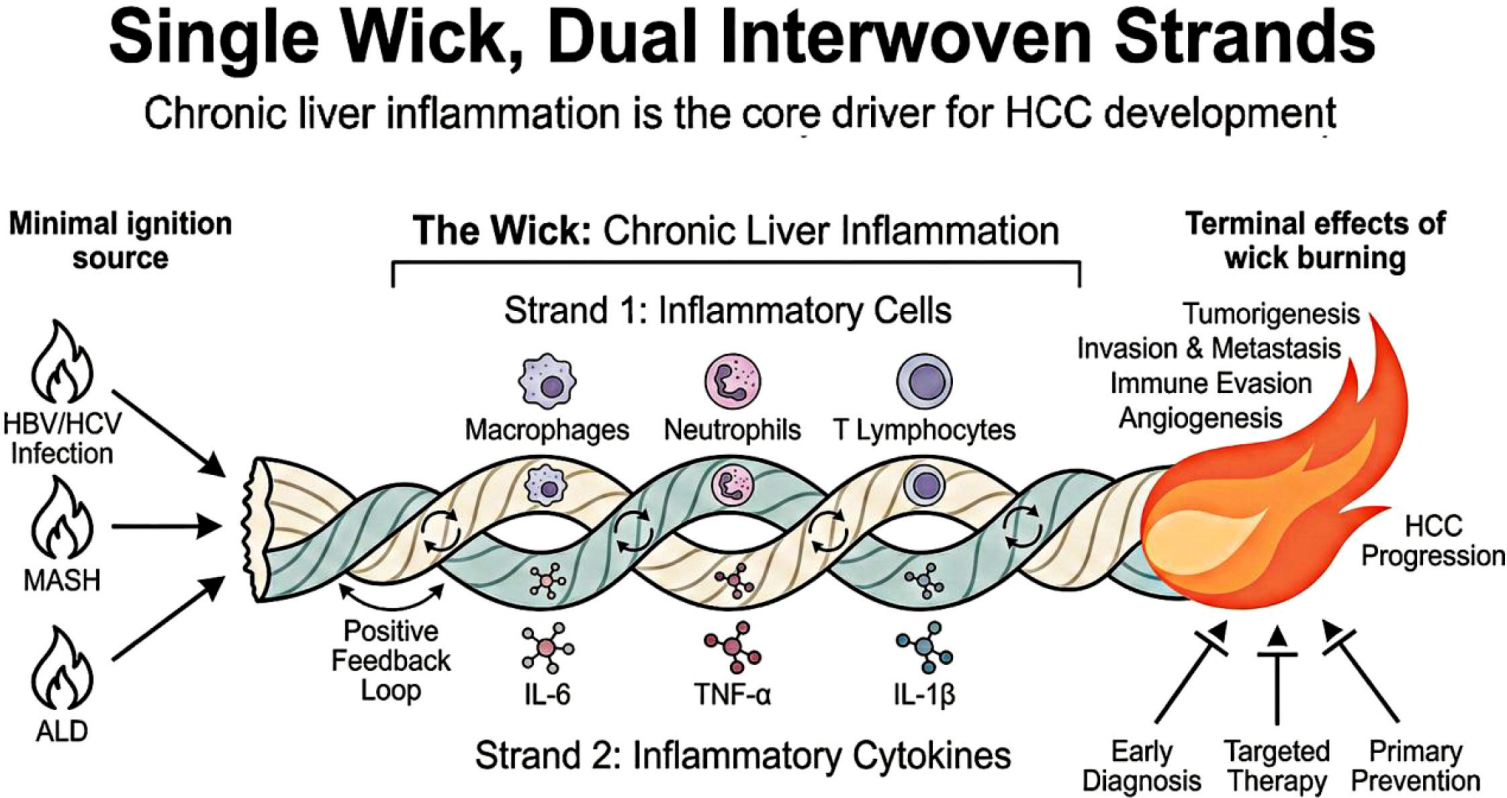
The “Single Wick, Dual Interwoven Strands” regulatory framework of inflammation-driven hepatocellular carcinoma progression.

Previous studies have mostly focused on the role of a single inflammatory cell or inflammatory cytokine in HCC, lacking a systematic and holistic interpretation of inflammation-driven HCC throughout the entire progression process. The core framework of “Single Wick, Dual Interwoven Strands” proposed in this review defines chronic inflammation as the “Wick” – the core driver of the entire continuum of HCC progression, while inflammatory cells and inflammatory cytokines are the two core functional carriers through which inflammation exerts its carcinogenic effects (the “two strands”). The synergistic interweaving of the dual strands drives five core terminal biological effects that run through the whole process of HCC malignant progression: epithelial-mesenchymal transition (EMT) and invasion/metastasis, tumor angiogenesis, immune escape, metabolic reprogramming, and therapeutic resistance. Based on this framework, this review systematically sorts out the molecular mechanisms of chronic inflammation-mediated HCC occurrence and development, dissects the interactive regulatory network between inflammatory cells and inflammatory cytokines, summarizes the HCC diagnostic and therapeutic strategies targeting inflammatory pathways and the bottlenecks in their clinical translation, and ultimately provides novel ideas and theoretical support for the precise prevention and control of HCC. **Figure 1** visually defines chronic liver inflammation as the core “Wick”, presents the two interwoven functional “Strands” (inflammatory cells and inflammatory cytokines), and illustrates how their synergistic interaction drives the five core hallmarks of HCC progression defined in this framework.

The core theoretical framework of this review: Chronic liver inflammation is defined as the “Wick”, the core driver of HCC progression. The two interwoven functional “Strands” are inflammatory cells and inflammatory cytokines, which form a positive regulatory loop and synergistically drive the whole process of HCC development, providing core targets for HCC prevention, diagnosis and treatment.

## 2 The Wick: chronic liver inflammation as the core driver of hepatocarcinogenesis

The liver is a vital metabolic and immune organ with a unique innate and adaptive immune cell network that mediates pathogen clearance and tissue injury repair. The gut-liver axis, defined by bidirectional crosstalk between the intestinal microbiome and the liver via the portal circulation, acts as a conserved upstream modulator of the inflammatory “Wick” across all HCC etiologies. Gut barrier dysfunction and microbiota dysbiosis occur consistently in HBV, HCV, MASH, and ALD, driving bacterial translocation and portal influx of microbiome-derived products (e.g., LPS) and metabolites (e.g., secondary bile acids, short-chain fatty acids). Specifically, microbiome-derived products such as LPS act as primary triggers that activate the TLR4 signaling in Kupffer cells (the first strand), subsequently inducing a burst of pro-inflammatory cytokines such as IL-6 and TNF-α (the second strand) to sustain the inflammatory ‘Wick’.

**Figure 2:**
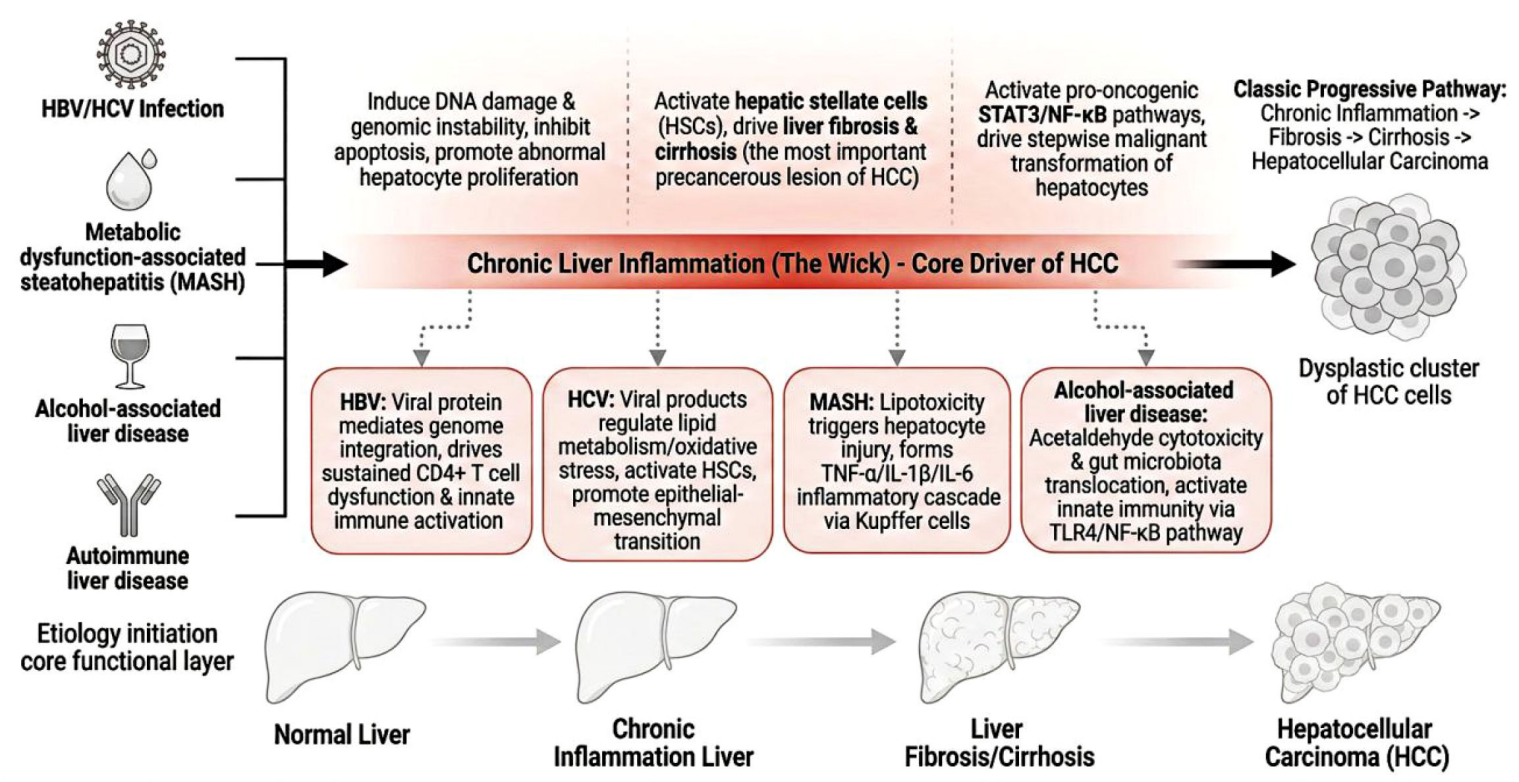
Core mechanisms of chronic inflammation-driven hepatocellular carcinoma tumorigenesis.

When the liver is subjected to persistent stimulation from viral infection, metabolic disorders, or alcohol toxicity, it initiates an innate immune response to eliminate damaging stimuli and repair damaged tissue (5). When harmful stimuli persist, acute inflammation fails to resolve, progressing to chronic non-resolving inflammation and forming a vicious cycle of “injury - inflammation - repair” that ultimately induces malignant transformation of hepatocytes (**Figure 2**) (6).

Regardless of etiology, HCC development follows the classic progressive pathway of “chronic inflammation - liver fibrosis - cirrhosis - liver cancer” (2). During this process, sustained inflammatory stimulation (the core Wick) drives two core preneoplastic events that establish the soil for hepatocarcinogenesis. First, chronic inflammation activates hepatic stellate cells (HSCs), promoting their transdifferentiation into myofibroblasts and massive extracellular matrix secretion, which drives progressive liver fibrosis and cirrhosis—the most important precancerous lesion for HCC (3, 4). This fibrosis-carcinoma sequence is the core pathological basis for the sustained Wick to drive malignant transformation, with irreversible stromal remodeling further amplifying the inflammatory cascade (5). Second, persistent oxidative stress and DNA damage in the inflammatory microenvironment directly induce hepatocyte gene mutations and genomic instability, while only briefly activating conserved core inflammatory signaling switches (NF-κB, STAT3) that prime hepatocytes for malignant transformation (7, 8).

Chronic liver inflammation caused by different etiologies has both conserved core regulatory pathways and etiology-specific molecular features in HCC tumorigenesis (9–11). Hepatitis B virus (HBV) infection mediates hepatocyte genome integration and epigenetic modification via viral proteins to induce chromosomal instability, while driving sustained inflammation characterized by CD4+ cytotoxic T cell dysfunction and aberrant innate immune activation (12). Hepatitis C virus (HCV) regulates hepatocyte lipid metabolism and oxidative stress via viral core protein, while activating HSCs to accelerate fibrosis progression and hepatocyte malignant transformation (13). Metabolic dysfunction-associated steatohepatitis (MASH)-related inflammation is initiated by lipotoxicity-induced hepatocyte injury and damage-associated molecular patterns (DAMPs) release, forming a TNF-α/IL-1β/IL-6 inflammatory cascade through Kupffer cells and recruited mononuclear macrophages (14). The core of chronic inflammation mediated by alcohol-associated liver disease (ALD) originates from the direct cytotoxicity of acetaldehyde, an alcohol metabolite, and sustained lipopolysaccharide exposure caused by gut microbiota translocation, which activates innate immune cells through the TLR4/NF-κB pathway (15). The core similarities and differences in inflammatory features across these etiologies are summarized in **Table 1**. Recent advances in single-cell and spatial transcriptomics have further revealed etiology-specific differences in the inflammatory microenvironment of HCC, providing novel targets for etiology-specific prevention and treatment (16).

**Table 1:** Etiology-specific inflammatory features of HCC and relative contributions of core regulatory elements.

Etiology	Dominant inflammatory cell subsets (First Strand)	Relative dominance of IL-6/STAT3 axis & core cytokine profile (Second Strand)	Key translational implications
HBV	Adaptive immune disorder: CD4+ cytotoxic T cell dysfunction, aberrant innate immune activation	Synergistic driver: IL-6/STAT3 axis is co-activated by viral proteins and the inflammatory cascade, with chronic upregulation of IL-6 and TNF-α; NF-κB acts as the upstream core switch	Antiviral therapy is the foundational intervention; IL-6/STAT3 targeting serves as an adjunctive strategy to reverse T cell dysfunction in chronic infection
HCV	Innate immune activation, persistent hepatic stellate cell activation, T cell exhaustion	Co-dominant driver: IL-6/STAT3 axis acts in parallel with the TGF-β-mediated fibrotic cascade, with sustained upregulation of IL-6, TNF-α and TGF-β	Dual targeting of IL-6/STAT3 and TGF-β pathways is a promising strategy to inhibit fibrosis-associated malignant transformation
MASH	Dominant Kupffer cell/recruited macrophage infiltration, robust neutrophil recruitment	Dominant core driver: IL-6/STAT3 axis shows the highest activation level across all etiologies, directly linking lipotoxicity to hepatocyte malignant transformation, with a TNF-α/IL-1β/IL-6 cascade as the core profile	IL-6/STAT3 targeting is a first-line candidate for MASH-HCC chemoprevention and treatment, with the most robust preclinical evidence
ALD	TLR4-mediated innate immune activation, prominent Kupffer cell activation, significant oxidative stress	Secondary amplifier: NF-κB is the dominant upstream driver; IL-6/STAT3 axis acts as a secondary amplifier of the inflammatory cascade downstream of TLR4 activation, with sustained TNF-α and IL-1β upregulation	TLR4/NF-κB targeting is the priority intervention; IL-6/STAT3 inhibition is adjunctive to reduce inflammatory amplification in advanced ALD
Conserved Commonality	Kupffer cell and hepatic stellate cell activation across all etiologies	NF-κB/STAT3 axis is the conserved core pathway in all HCC subtypes, with etiology-specific functional dominance	Pan-inflammation targeting strategies are universally applicable, while etiology-specific regimen optimization is required for maximal efficacy

HBV, hepatitis B virus; HCV, hepatitis C virus; MASH, metabolic dysfunction-associated steatohepatitis; ALD, alcohol-associated liver disease; HCC, hepatocellular carcinoma; IL, interleukin; TNF-α, tumor necrosis factor-α; TGF-β, transforming growth factor-β.

The classic progressive pathological pathway of HCC: normal liver → chronic liver inflammation → liver fibrosis/cirrhosis → HCC. It illustrates the core mechanisms by which sustained chronic inflammation drives hepatocyte malignant transformation through promoting fibrosis and inducing genomic instability.

## 3 The dual interwoven strands: two core functional carriers of inflammation-driven HCC

**Figure 3:**
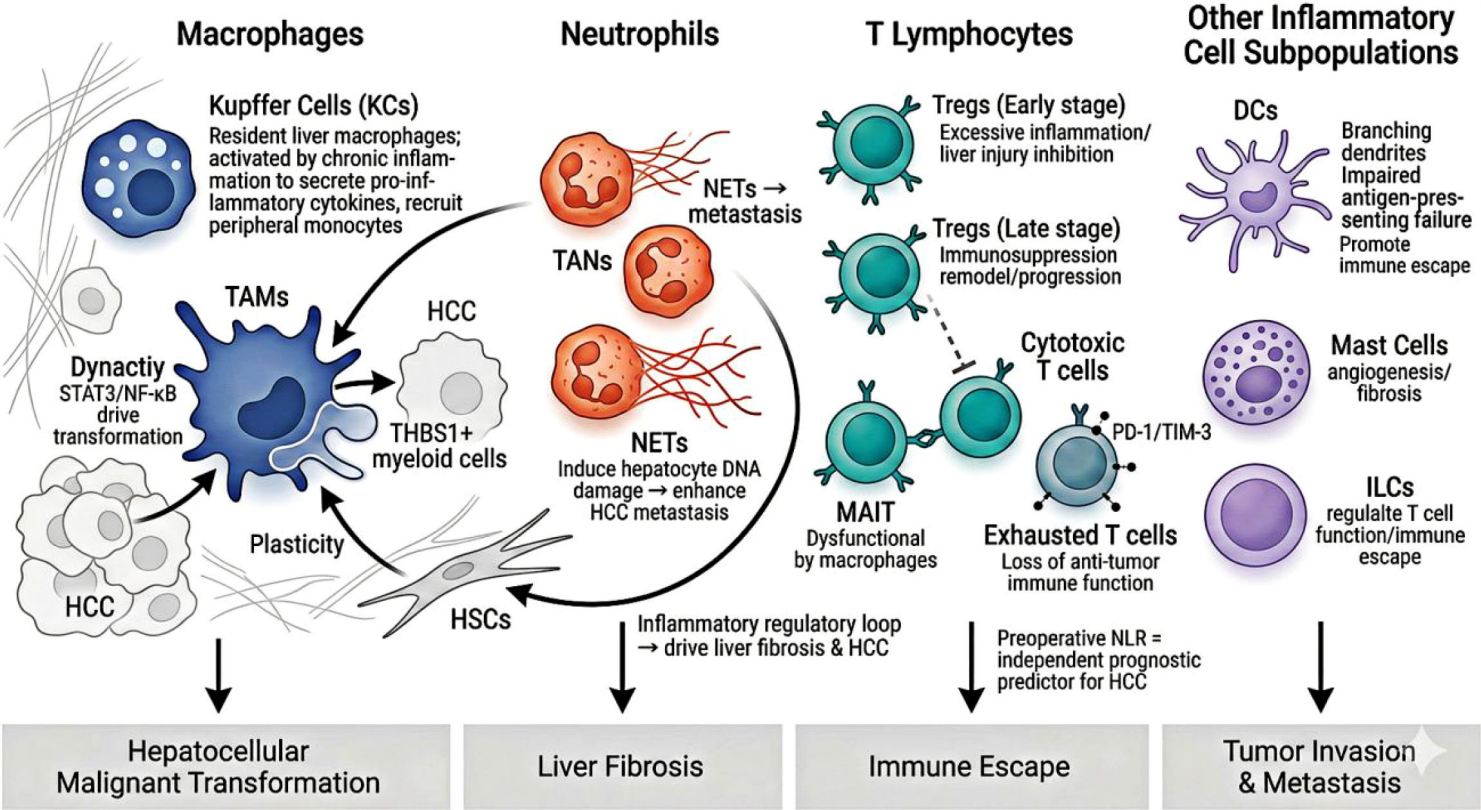
Core inflammatory cell subsets in the hepatocellular carcinoma microenvironment.

As the core Single Wick driving the entire HCC progression continuum, the pro-carcinogenic effects of chronic inflammation are exclusively executed via two interwoven functional carriers: the first “Strand” – inflammatory cells (including macrophages, neutrophils, and T lymphocytes), and the second “Strand” – inflammatory cytokines (represented by IL-6, TNF-α, and IL-1β). These two components constitute the dual interwoven strands of inflammation-driven HCC: while each exerts independent pro-tumorigenic effects, their tight reciprocal regulation forms cascade-amplified inflammatory loops that drive HCC initiation, proliferation, invasion, metastasis, and immune evasion (**Figure 3**).

### 3.1 The first “strand”: inflammatory cells – core regulators of the hepatocellular carcinoma microenvironment

Inflammatory cells are one of the most abundant cell populations in the tumor microenvironment (TME) of HCC, as well as the primary executors of the pro-tumorigenic effects of chronic inflammation (1). Different subpopulations of inflammatory cells exert heterogeneous functions in HCC progression, including pro-inflammatory/pro-tumor cell subpopulations that directly drive malignant transformation of hepatocytes and promote tumor proliferation and metastasis, as well as cell subpopulations that exert anti-tumor immune effects. The persistence of chronic inflammation breaks this balance, polarizing the TME towards an immunosuppressive and pro-tumor direction (16).

#### 3.1.1 Macrophages: the core hub of the inflammatory microenvironment

Macrophages are the most abundant innate immune cells in the liver, including resident Kupffer cells (KCs) and peripherally recruited tumor-associated macrophages (TAMs), and serve as the core hub linking the inflammatory “Wick” to the dual functional strands in our framework (17). Under chronic inflammatory stimulation, KCs are activated to secrete pro-inflammatory cytokines and chemokines, recruiting circulating monocytes to the liver and driving their differentiation into TAMs. The inflammatory microenvironment further induces M2-like polarization of TAMs, which acquire an immunosuppressive, pro-angiogenic and pro-metastatic phenotype, and directly activate pro-oncogenic STAT3 and NF-κB signaling in hepatocytes via cytokine secretion to drive malignant transformation (18, 19).

Recent single-cell and spatial transcriptomic studies have revealed the high functional heterogeneity of hepatic macrophage subsets. Shifts in the inflammatory macrophage niche drive KCs to switch from a homeostatic clearance phenotype to a pro-tumor state, further amplifying the hepatic inflammatory cascade (17). As the core bridge between innate and adaptive immunity, macrophages also mediate tumor immune escape via crosstalk with T cells: TAMs induce effector T cell exhaustion, promote regulatory T cell recruitment, and reshape the immunosuppressive microenvironment, forming a key component of the interactive regulatory network between the two “strands” of our framework (20).

#### 3.1.2 Neutrophils: plastic regulators and amplifiers of the inflammatory cascade

Neutrophils are the most abundant leukocytes in peripheral blood and act as the first line of innate immune defense in the liver, with well-documented functional plasticity and a context-dependent “double-edged sword” role in liver inflammation and tumorigenesis, consistent with the bidirectional nature of inflammatory responses outlined in Section 6. In the setting of acute resolving liver inflammation, neutrophils exert tissue-protective and anti-tumor effects: they rapidly infiltrate the injured liver to clear invading pathogens, remove damaged hepatocytes and pre-malignant cells with genomic instability, and initiate physiological tissue repair, thereby inhibiting the initiation of hepatocarcinogenesis. This anti-tumor phenotype, defined as the N1 phenotype of tumor-associated neutrophils (TANs), is characterized by high expression of pro-inflammatory and immunostimulatory cytokines (e.g., TNF-α, IL-12), enhanced antigen presentation capacity, and direct cytotoxicity against pre-malignant and malignant hepatocytes.

However, in the context of chronic non-resolving liver inflammation (the core “Wick” of our framework), sustained inflammatory stimulation drives persistent neutrophil infiltration into the liver parenchyma and tumor tissue, promoting their polarization towards the immunosuppressive and pro-tumor N2 phenotype, which act as key amplifiers of the inflammatory cascade and critical drivers of HCC progression (21). N2-polarized TANs drive HCC development through multiple core mechanisms. First, they can directly induce DNA damage and malignant transformation of hepatocytes by releasing neutrophil extracellular traps (NETs), while further amplifying the inflammatory response through the secretion of pro-inflammatory cytokines and chemokines, thereby promoting HCC invasion and metastasis (22–25). A study by Yang et al. confirmed that increased NETs in the HCC microenvironment significantly enhance the metastatic potential of HCC cells by triggering a tumorous inflammatory response (26). Second, neutrophils can activate hepatic stellate cells (HSCs) and macrophages by releasing inflammatory mediators, forming a “neutrophil-macrophage-HSC” inflammatory regulatory loop that drives the progression of liver fibrosis—the most important precancerous lesion of HCC—and further amplifies the chronic inflammatory cascade (21). In addition, the preoperative peripheral blood neutrophil-to-lymphocyte ratio (NLR), a systemic marker of neutrophil-mediated chronic inflammation, has been validated as an independent predictor of postoperative recurrence, treatment response and long-term survival prognosis in HCC patients, further corroborating the central role of pro-tumor neutrophils in HCC progression (27).

#### 3.1.3 T lymphocytes: dual regulators of inflammation and immune escape

T lymphocytes are the core cells of adaptive immunity, and the imbalance of T cell subpopulations in the inflammatory microenvironment of HCC is a key link mediating immune escape and tumor progression (24). Chronic liver inflammation persistently activates effector T cells, while long-term antigen stimulation induces T cell exhaustion, which is characterized by high expression of immune checkpoint molecules such as PD-1 and TIM-3, leading to the loss of anti-tumor immune function. Meanwhile, the inflammatory microenvironment induces the recruitment and activation of regulatory T cells (Tregs), further inhibiting the function of effector T cells and forming an immunosuppressive tumor microenvironment (25, 28, 29).

Using single-cell sequencing technology, He et al. systematically dissected the phenotypic heterogeneity of human CD127+ innate lymphoid cells in HCC tissues and found that they participate in the immune escape process of HCC by secreting inflammatory cytokines to regulate T cell function (30). A study by Patseas et al. further revealed that the interaction between myeloid cells and T cells in chronic liver inflammation is a core link driving the progression from liver fibrosis to HCC (21). In addition, inflammatory cytokines such as IFN-γ secreted by cytotoxic T cells exert anti-tumor effects in the early stage of inflammation, but under sustained stimulation of chronic inflammation, they induce the expression of immune checkpoint molecules, which in turn promotes the occurrence of immune escape (31). The spatiotemporal dynamic changes of different T cell subpopulations in the HCC inflammatory microenvironment further highlight the complexity of the T cell regulatory network: Wang et al. demonstrated that regulatory T cells (Tregs), a key immunosuppressive T cell subset, have a “double-edged sword” role in MASH-to-HCC progression (32). In early inflammatory stages, Tregs mitigate liver injury by suppressing excessive inflammation; in established tumors, they promote tumor proliferation and immune escape by remodeling the immunosuppressive microenvironment. Dynamic shifts in Treg infiltration density and functional phenotype are closely associated with HCC patient prognosis. Using spatial profiling technology, Ruf et al. further confirmed that at the invasive margin of HCC, tumor-associated macrophages can induce dysfunction of mucosal-associated invariant T (MAIT) cells, causing them to lose their killing activity against tumor cells and switch to a pro-inflammatory phenotype. This functional remodeling of local T cell subpopulations is most prominent at the tumor invasive front, serving as a key spatiotemporal feature driving tumor progression (33).

#### 3.1.4 Other inflammatory cell subpopulations

In addition to the core cell subpopulations mentioned above, other inflammatory cell subpopulations including dendritic cells (DCs), mast cells, and innate lymphoid cells (ILCs) also play important regulatory roles in the inflammatory microenvironment of HCC. As the most potent antigen-presenting cells in the body, DCs have impaired antigen-presenting function in chronic inflammation, which prevents them from effectively activating T cell-mediated anti-tumor immunity, thereby promoting immune escape of HCC (34). Mast cells can regulate angiogenesis and fibrosis in the inflammatory microenvironment by secreting histamine, pro-inflammatory cytokines, and other mediators, participating in the progression of HCC (35).

The key inflammatory cell subsets (the first “Strand” of the framework) in the HCC tumor microenvironment, including macrophages, neutrophils, T lymphocytes and other functional cell populations, and clarifies their core roles in driving HCC progression.

### 3.2 The second “strand”: inflammatory cytokines – direct regulatory molecules of malignant phenotypes in hepatocellular carcinoma

**Figure 4:**
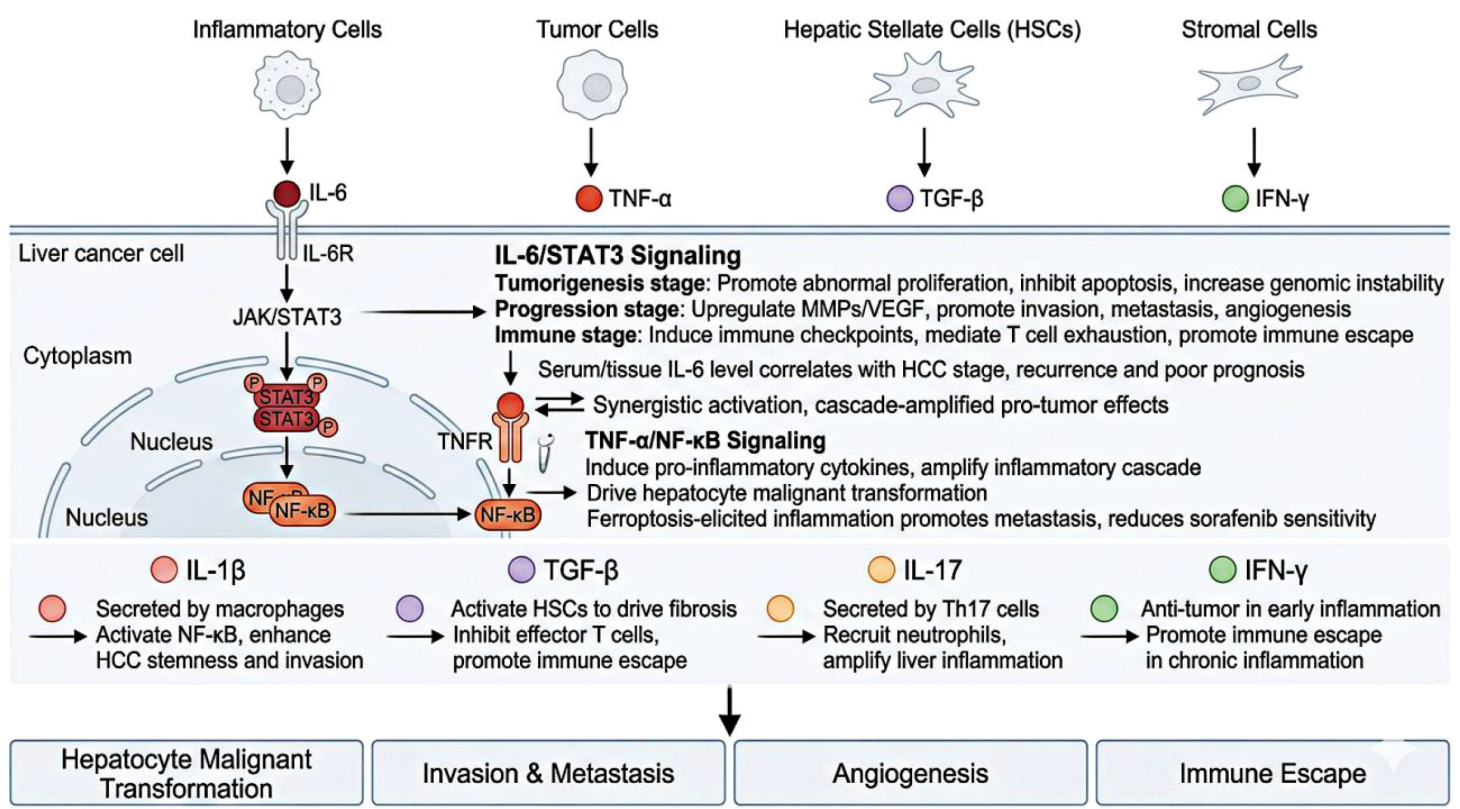
Core inflammatory cytokines and signaling pathways in hepatocellular carcinoma progression.

Inflammatory cytokines are small polypeptides secreted by inflammatory cells, tumor cells, and stromal cells, serving as the direct molecular executors of chronic inflammation’s pro-tumor effects and the core signaling bridge linking inflammatory cells and tumor cells (32). In the HCC microenvironment, pro-inflammatory cytokines centered on IL-6 and TNF-α directly regulate tumor cell malignant phenotypes by activating the two-core conserved signaling axes introduced in Section 2: STAT3 and NF-κB. Meanwhile, they reshape the inflammatory microenvironment to form a positive feedback loop of “inflammatory cytokines - tumor cells - inflammatory cells” (**Figure 4**) (33).

#### 3.2.1 IL-6/STAT3 signaling pathway: the core axis of inflammation-driven hepatocellular carcinoma

As delineated in **Table 1**, the functional output and therapeutic potential of the IL-6/STAT3 axis vary significantly across different HCC etiologies, in line with its etiology-specific relative dominance. IL-6 is the central inflammatory cytokine linking chronic inflammation to HCC initiation and progression, mainly secreted by activated macrophages, neutrophils, and fibroblasts. It exerts broad pro-tumor effects by binding to its receptor and activating the downstream JAK/STAT3 signaling pathway (19). In the tumorigenesis stage, IL-6/STAT3 activation promotes hepatocyte proliferation, inhibits apoptosis, and increases genomic instability to drive malignant transformation (35). In the progression stage, this pathway upregulates MMPs and VEGF to promote HCC invasion, metastasis, and angiogenesis (36), and induces immune checkpoint molecule expression to mediate T cell exhaustion and immune escape (37).

Recent studies have confirmed that nuclear protein Pirin promotes HCC initiation and progression by enhancing IL-6/STAT3 pathway activation (19), while IL-6 trans-signaling drives malignant transformation of liver progenitor cells (35). Clinical studies have verified that elevated IL-6 expression in peripheral blood and tumor tissues of HCC patients is closely associated with advanced tumor stage, recurrence risk, and poor prognosis (38).

#### 3.2.2 TNF-α/NF-κB signaling pathway: the core switch of the inflammatory cascade

TNF-α, mainly secreted by activated macrophages and T cells, is another core pro-inflammatory cytokine. Its downstream NF-κB signaling pathway is the core molecular switch of the inflammatory cascade, and a key link between chronic inflammation and HCC (39). In chronic liver inflammation, TNF-α-mediated NF-κB activation induces the expression of a broad panel of pro-inflammatory cytokines and chemokines to amplify the inflammatory cascade, while directly regulating hepatocyte survival and proliferation to drive malignant transformation (40, 41).

Studies have confirmed that sustained NF-κB activation is a core mechanism driving hepatocyte apoptosis, inflammatory cascade amplification, and hepatocarcinogenesis, and targeted inhibition of this pathway can block hepatocyte malignant transformation and delay HCC progression (42). Recent research found that ferroptosis-induced inflammation upregulates prometastatic factors via activating the TNF-α/NF-κB axis, promoting HCC metastasis and reducing sorafenib sensitivity. Notably, the NF-κB and STAT3 pathways form a synergistically activated interactive regulatory network in the HCC microenvironment: they amplify the cascade release of IL-6 and TNF-α and jointly drive the acquisition of malignant phenotypes in hepatocytes, producing synergistically amplified pro-tumor effects (43–45). This synergistic activation also mediates metabolic reprogramming of HCC cells, providing an energy basis for tumor proliferation and forming a positive feedback loop of “inflammatory cytokines - metabolic reprogramming - tumor progression” (46–48).

#### 3.2.3 Other key inflammatory cytokines

In addition to IL-6 and TNF-α, other inflammatory cytokines including IL-1β, TGF-β, IFN-γ, and IL-17 also play important roles in the progression of HCC. IL-1β is mainly secreted by activated macrophages and can promote the formation of an inflammatory microenvironment by activating the NF-κB pathway, while enhancing the stemness and invasive ability of HCC cells (49). TGF-β is a core cytokine mediating liver fibrosis and HCC immunosuppression, which can promote the progression of liver fibrosis by activating HSCs, while inhibiting the function of effector T cells and promoting immune escape of HCC (50). IL-17 is mainly secreted by Th17 cells and can amplify the inflammatory response in the liver by recruiting neutrophil infiltration, driving the initiation and progression of HCC (51).

The key inflammatory cytokines (the second “Strand” of the framework) and their mediated core signaling pathways in HCC progression, mainly including the IL-6/STAT3 and TNF-α/NF-κB axes, and their core pro-tumor effects in HCC development.

## 4 Interweaving of the dual strands: interactive regulatory network driving HCC malignant progression

**Figure 5:**
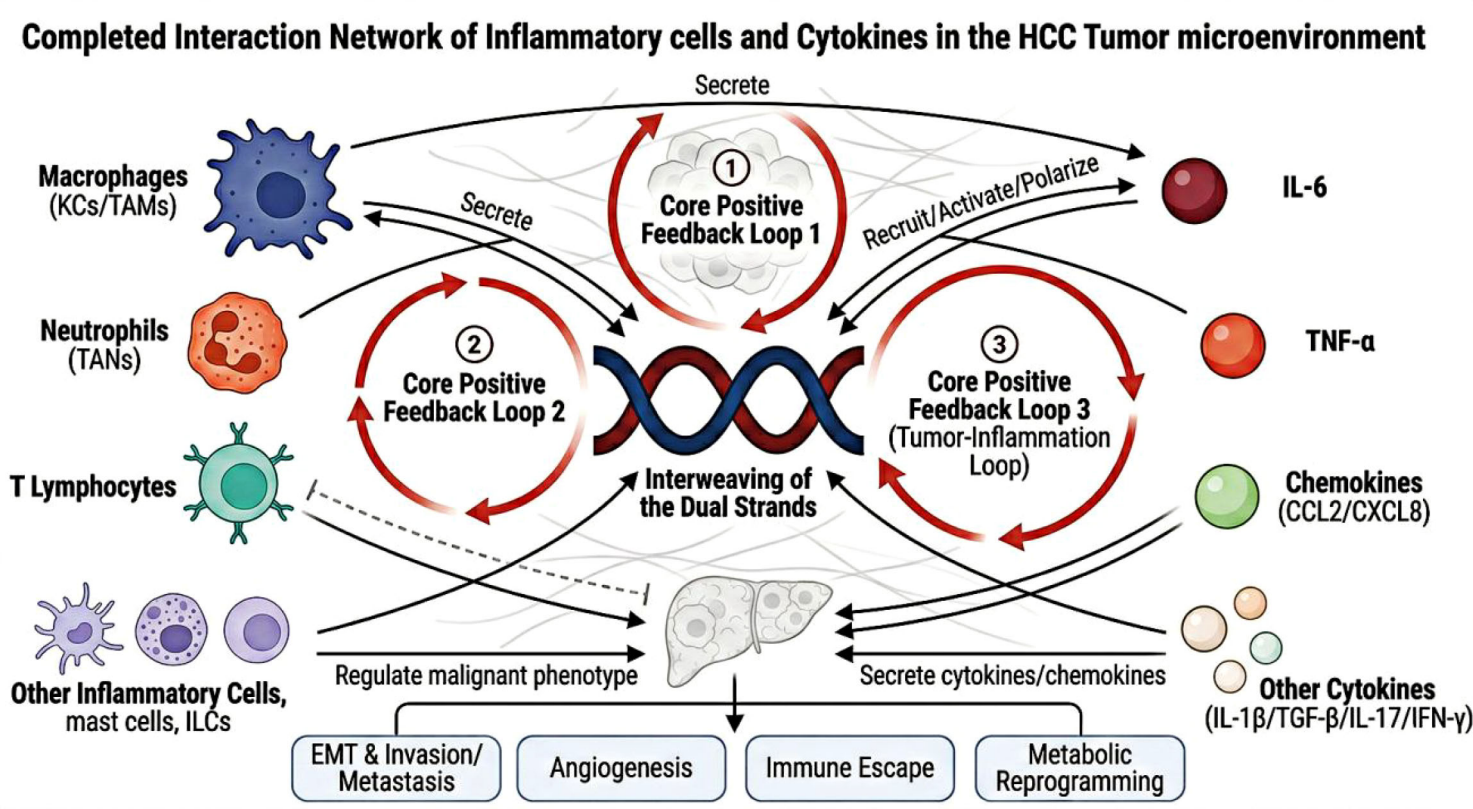
Interactive network between inflammatory cells and cytokines in hepatocellular carcinoma.

Inflammatory cells and inflammatory cytokines do not act independently. Instead, there is tight interactive regulation between them, forming a complex inflammatory regulatory network that exerts cascade-amplified effects in the initiation and progression of HCC, driving the entire progression of HCC from precancerous lesions to advanced metastasis throughout the whole process (52). This interactive regulation is mainly reflected in three levels: first, inflammatory cells reshape the inflammatory microenvironment and regulate the malignant phenotype of tumor cells by secreting inflammatory cytokines; second, inflammatory cytokines further amplify the inflammatory cascade by regulating the polarization, recruitment, and activation of inflammatory cells; third, tumor cells can reversely regulate the inflammatory microenvironment by secreting inflammatory cytokines and chemokines, forming a positive feedback pro-tumor loop of “tumor - inflammation” (**Figure 5**) (53).

### 4.1 Interactive regulatory loop between inflammatory cells and inflammatory cytokines

The core of the “interweaving of the dual strands” lies in the positive feedback regulatory loop formed by inflammatory cells and cytokines, which realizes cascade amplification of the inflammatory signal, rather than the independent effect of a single cell or cytokine. On the one hand, activated inflammatory cells (the first “strand”) secrete a variety of pro-inflammatory cytokines and form the material basis of the second “strand”; on the other hand, inflammatory cytokines (the second “strand”) in turn regulate the recruitment, polarization and functional activation of inflammatory cells, continuously expand the pro-tumor inflammatory cell pool, and form a closed-loop cascade amplification effect (54).

This interactive loop further forms a “tumor-inflammation” positive feedback circuit with tumor cells: inflammatory signals drive tumor cells to secrete chemokines and pro-inflammatory factors, which in turn continuously recruit and activate inflammatory cells to reshape the pro-tumor microenvironment and form a self-sustaining inflammatory cascade that continuously drives HCC progression (55–57).

### 4.2 Core biological effects of the inflammatory network driving malignant progression of hepatocellular carcinoma

The “interwoven dual strands” inflammatory network exerts a synergistic cascade amplification effect that cannot be achieved by a single cell or cytokine and drives the entire malignant progression of HCC through five core biological processes, which are the terminal biological outcomes of the “Single Wick, Dual Interwoven Strands” framework (54).

#### 4.2.1 Driving EMT and invasion/metastasis

The inflammatory network formed by the interweaving of inflammatory cells and cytokines is the core upstream regulatory signal driving EMT of HCC cells. Integrated findings from multiple studies have confirmed that the network synergistically activates multiple pro-oncogenic signaling pathways including STAT3, NF-κB and Smad, upregulates EMT-related transcription factors, and cooperates with matrix-degrading enzymes secreted by inflammatory cells to jointly enhance the invasive and metastatic potential of HCC cells (58–60). Latest spatial transcriptomic studies have further mapped the spatial distribution pattern of the interwoven strands at the HCC invasive front, revealing that tumor-associated macrophages (Strand 1) form a tight spatial co-localization niche with invasive HCC cells, and secrete high levels of IL-6 and TGF-β (Strand 2) *in situ* to directly drive EMT program activation in adjacent tumor cells (61). This is the core terminal effect of the cascade amplification of the inflammatory network and is also a key mechanism leading to poor prognosis of patients.

#### 4.2.2 Promoting tumor angiogenesis

The interwoven inflammatory network forms positive feedback pro-angiogenic loop through the multi-cellular interaction between inflammatory cells, cytokines and tumor cells. The network synergistically upregulates the expression of multiple pro-angiogenic factors including VEGF and bFGF and forms a specific interactive niche with vascular endothelial cells at the tumor invasive front (62–66). Latest single-cell and spatial transcriptomic joint analysis has precisely resolved this pro-angiogenic niche at single-cell resolution, revealing that TAMs and TANs (Strand 1) are the core cellular components of the perivascular niche, and their secreted IL-8, TNF-α and VEGF (Strand 2) form a local cytokine gradient to directly promote vascular endothelial cell proliferation and tube formation, providing direct *in situ* evidence for the pro-angiogenic effect of the interwoven strands (62). This continuous pro-angiogenic effect provides necessary material conditions for the sustained growth and distant metastasis of HCC.

#### 4.2.3 Mediating tumor immune escape

The inflammatory network is the core driving force for the formation of the immunosuppressive microenvironment of HCC. Accumulating evidence confirms that the interwoven inflammatory cell-cytokine network regulates multiple key steps of the anti-tumor immune response (64, 65). Latest spatial transcriptomic and single-cell sequencing studies have directly visualized the *in situ* crosstalk between inflammatory cells and cytokines in the HCC microenvironment, and verified this network is the core driver of T cell exhaustion and regional immune suppression (67, 68). It drives effector T cell exhaustion, promotes the activation and infiltration of immunosuppressive cell subsets, and upregulates immune checkpoint molecule expression on tumor cells. This multi-dimensional regulation ultimately mediates systemic immune escape of HCC and is a core cause of the limited efficacy of immunotherapy.

#### 4.2.4 Regulating tumor metabolic reprogramming

There is a bidirectional regulatory interaction between the interwoven inflammatory network and tumor metabolic reprogramming, which forms a self-amplifying “inflammation-metabolism” positive feedback loop (69, 70). Single-cell multi-omics and spatial metabolomics studies have verified the synergistic effect of the inflammatory cell-cytokine network on driving HCC metabolic reprogramming at the single-cell and spatial level (71, 72). Synergistic activation of STAT3 and NF-κB signaling by the inflammatory network drives HCC metabolic reprogramming, allowing tumor cells to adapt to hypoxic, nutrient-poor microenvironments. In turn, metabolites from tumor metabolic reprogramming (e.g., lactic acid) reshape the inflammatory network to further amplify the pro-tumor inflammatory cascade (73, 74).

#### 4.2.5 Mediating therapeutic resistance

The interwoven inflammatory network is a core driver of primary and acquired resistance to first-line systemic therapies for HCC, a key terminal biological effect of our “Single Wick, Dual Interwoven Strands” framework. Latest spatial transcriptomic studies have identified a distinct drug-resistant niche at the tumor-stroma interface, where the interwoven strands are significantly enriched, providing direct *in situ* evidence for the role of the inflammatory network in mediating therapeutic resistance (67). For sorafenib and lenvatinib, the two strands synergistically mediate resistance through multi-layered mechanisms: tumor-associated macrophages (TAMs) and neutrophils (TANs, the first strand) secrete high levels of IL-6 and TNF-α (the second strand), which activate STAT3/NF-κB and ERK signaling cascades to counteract the anti-tumor effects of these agents, while driving metabolic reprogramming and epithelial-mesenchymal transition to further reduce drug sensitivity. Notably, sorafenib-resistant HCC cells with sustained ERK phosphorylation further upregulate PD-1 expression, forming a bidirectional link between targeted therapy resistance and immune checkpoint activation, which bridges the two core therapeutic resistance pathways in HCC (37, 48).

For anti-PD-1/PD-L1 immune checkpoint blockade, the interwoven inflammatory network is a central mediator of primary and acquired treatment failure. Immunosuppressive inflammatory cell subsets (the first strand), including M2-like TAMs, regulatory T cells and TANs, form an immune-excluded barrier at the tumor margin to block effector T cell infiltration and function. Concurrently, sustained high levels of IL-6, TGF-β and TNF-α (the second strand) induce persistent effector T cell exhaustion and upregulate immune checkpoint molecule expression on both tumor cells and immune cells, driving the formation of an immunosuppressive microenvironment and primary resistance to immunotherapy (20, 64). Spatial transcriptomic studies have further identified that stromal-inflammatory crosstalk forms distinct immune-excluded niches, which are independent drivers of immunotherapy resistance and post-treatment recurrence in HCC patients (33, 62).

### 4.3 Non-immune stromal components: critical amplifiers of the “dual interwoven strands” inflammatory network

We explicitly define hepatic stellate cells (HSCs), cancer-associated fibroblasts (CAFs), and extracellular matrix (ECM) as critical signal amplifiers independent of the two core strands, with no functional overlap with the core pro-tumor execution roles of inflammatory cells (Strand 1) and cytokines (Strand 2). They amplify the “Strand 1-Strand 2” positive feedback loop at 3 non-redundant levels:

#### 4.3.1 Cascade amplification

Activated HSCs and CAFs secrete large amounts of chemokines to recruit and expand the pro-tumor inflammatory cell pool (Strand 1), while sustainably releasing core pro-inflammatory and profibrotic cytokines (IL-6, TNF-α, TGF-β) to stabilize and amplify Strand 2 signal output, breaking the limitation of local transient inflammatory response and forming a self-sustaining inflammatory cascade (4, 5, 14). Meanwhile, the activated stromal compartment directly promotes the stepwise progression of the “liver fibrosis-cirrhosis-HCC” axis, which is the core preneoplastic soil for the inflammatory Wick to drive malignant transformation (75).

#### 4.3.2 Spatial amplification

Remodeled ECM chelates inflammatory cytokines to form local high-concentration signal niches at the tumor invasive front, while enhanced matrix stiffness further promotes the pro-tumor polarization of Strand 1 cells (including M2-like TAMs and TANs) via integrin-mediated mechanotransduction, significantly improving the pro-tumor efficiency of the dual strands (76). Single-cell and spatial transcriptomic studies have further revealed that lineage-specific fibroblast subtypes drive heterogeneous ECM remodeling, forming distinct immune-excluded niches that directly block the anti-tumor immune response (77).

Wick sustaining amplification: Irreversible stromal remodeling mediated by chronic activation of HSCs/CAFs blocks physiological inflammation resolution, converting transient inflammatory stimuli into a sustained, non-resolving chronic inflammatory “Wick”. This persistent stromal-inflammatory crosstalk further drives hepatocyte genomic instability and malignant transformation, establishing a vicious cycle that continuously fuels HCC initiation and progression (5, 75). This amplifier role explains the limited efficacy of single-target therapy against the dual strands and provides novel synergistic targets for combination strategies (detailed in Section 5).

The “interweaving of the dual strands” core connotation of the framework, showing the positive feedback regulatory loops between inflammatory cells and cytokines, and the core biological effects of this interactive network in driving HCC malignant progression.

## 5 Targeting the Wick and strands: diagnostic, therapeutic, and preventive strategies for HCC

Based on the core driving role of inflammation in HCC initiation, progression, and therapeutic resistance (detailed in Section 4.2.5), targeting inflammatory pathways has become a pivotal research direction for the prevention, diagnosis, and treatment of HCC. In recent years, with the in-depth dissection of the mechanisms of inflammation-driven HCC, a large number of inflammation-targeted diagnostic and therapeutic strategies have been translated from basic research to clinical practice, providing novel tools for the precise prevention and control of HCC (68).

### 5.1 Inflammation-based biomarkers for early diagnosis and prognosis assessment of hepatocellular carcinoma

Chronic inflammation-related biomarkers are widely explored for the early diagnosis, risk stratification, and prognosis assessment of HCC. Peripheral blood systemic inflammatory markers, including NLR, platelet-to-lymphocyte ratio (PLR), and systemic immune-inflammation index (SII), can non-invasively reflect the host inflammatory state, and have been validated as independent predictors for postoperative recurrence, treatment response, and survival outcomes in HCC patients (78, 79). The core clinical application value of these markers is summarized in **Table 2**.

**Table 2:** Summary of inflammation-based biomarkers for HCC diagnosis and prognosis assessment.

Biomarker category	Biomarker	Full name/Definition	Core clinical application value
Systemic Inflammatory Markers	NLR	Neutrophil-to-Lymphocyte Ratio	Independent predictor of postoperative recurrence, systemic treatment response and long-term survival of HCC patients (78, 79)
Systemic Inflammatory Markers	PLR	Platelet-to-Lymphocyte Ratio	Non-invasive assessment of systemic inflammatory state, auxiliary prediction of HCC prognosis and treatment response (79, 81)
Systemic Inflammatory Markers	SII	Systemic Immune-Inflammation Index	Improves the discrimination of HCC prognostic prediction models, used for postoperative risk stratification of HCC patients (79, 83)
Inflammatory Cytokine & Chemokine Markers	IL-6	Interleukin-6	Correlates with HCC stage, postoperative recurrence risk and targeted therapy response; used for auxiliary diagnosis and prognosis assessment (41)
Inflammatory Cytokine & Chemokine Markers	TNF-α	Tumor Necrosis Factor-α	Core marker reflecting the inflammatory cascade activity, predicts the progression and treatment response of HCC (41)
Inflammatory Cytokine & Chemokine Markers	CXCL5/CXCL10/MIP-3α	C-X-C Motif Chemokine Ligand 5/10/Macrophage Inflammatory Protein-3α	Effective for early detection of HCC, accurately indicates microvascular invasion status and tumor progression degree (80)

HCC, hepatocellular carcinoma; NLR, Neutrophil-to-Lymphocyte Ratio; PLR, Platelet-to-Lymphocyte Ratio; SII, Systemic Immune-Inflammation Index; IL-6, Interleukin-6; TNF-α, Tumor Necrosis Factor-α.

Limitations and clinical optimization strategies: Despite their convenience, these systemic inflammatory markers have notable inherent limitations. Their specificity and sensitivity for HCC remain modest, with most studies reporting AUROC values of 0.6–0.75 for prognostic stratification (78–81). They are also vulnerable to confounding by non-tumor inflammatory conditions (e.g., active hepatitis, infection, post-procedural inflammation), lack universal standardized cut-off values, and show variable predictive performance across HCC of different etiologies (82, 83).

To address these gaps, integrated multi-modal strategies are proposed to enhance their clinical utility, aligned with our core framework. First, combining NLR/PLR/SII with traditional HCC biomarkers (AFP, PIVKA-II) significantly improves the accuracy of early detection and risk stratification. Second, integrating these markers with circulating inflammatory cytokines (e.g., IL-6, the core of the second “strand”) or ctDNA molecular signatures can refine patient stratification by capturing both systemic and tumor-intrinsic inflammatory features. Third, combination with imaging approaches (e.g., gadoxetic acid-enhanced MRI radiomics) enables preoperative prediction of microvascular invasion by integrating systemic inflammation with *in situ* tumor phenotypes.

### 5.2 Inflammation-targeted therapeutic strategies for hepatocellular carcinoma

In addition to directly targeting the two core strands, strategies targeting stromal components (the key amplifiers of the inflammatory network) have also emerged as promising complementary approaches for HCC treatment. At present, inflammation-targeted therapeutic strategies for HCC mainly include three categories: small molecule inhibitors of inflammatory pathways, inflammation-targeted biological agents, and combination regimens of inflammation targeting combined with immunotherapy (**Table 3**) (78).

**Table 3:** Summary of inflammation-targeted therapeutic strategies for hepatocellular carcinoma.

Therapeutic category	Representative agents	Core targets	Mechanism of action
Small molecule inhibitors	JAK inhibitors (Ruxolitinib, Baricitinib)	JAK1/2, IL-6/JAK/STAT3 axis	Block IL-6-mediated STAT3 activation, inhibit tumor cell proliferation and metastasis, remodel immunosuppressive TME (85, 86)
Small molecule inhibitors	NF-κB inhibitors (BAY 11-7082, DHMEQ)	NF-κB signaling pathway	Inhibit inflammatory cascade amplification, block hepatocyte malignant transformation, delay liver fibrosis and HCC progression (44, 84)
Small molecule inhibitors	MAPK inhibitors	MAPK/ERK signaling pathway	Counteract targeted therapy resistance, inhibit tumor cell survival and invasion (48, 85)
Biological agents	Tocilizumab	IL-6 receptor (IL-6R)	Block IL-6/STAT3 signaling pathway, inhibit tumor proliferation, enhance ICI efficacy (88)
Biological agents	Anti-TNF-α antibodies (Infliximab, Adalimumab)	TNF-α	Inhibit TNF-α/NF-κB inflammatory cascade, alleviate chronic hepatic inflammation (87)
Biological agents	Anti-CSF1R antibodies (Pexidartinib)	CSF1R	Deplete TAMs, reverse M2-like polarization, restore anti-tumor immunity (92, 93)
Combination regimens	JAK/STAT3 inhibitors + ICIs	JAK1/2, PD-1/PD-L1	Reverse immunosuppressive TME, restore effector T cell function, enhance ICI anti-tumor efficacy (85, 92)
Combination regimens	CSF1R inhibitors + ICIs	CSF1R, PD-1/PD-L1	Deplete immunosuppressive TAMs, promote T cell infiltration into tumor tissues (92, 93)
Combination regimens	Stereotactic body radiotherapy + Sintilimab + Bevacizumab	Multiple inflammatory pathways, VEGF, PD-1	Radiotherapy induces immunogenic cell death, synergizes with anti-angiogenic and immune checkpoint blockade (94)
Combination regimens	Bavituximab + Pembrolizumab	Phosphatidylserine, PD-1	Target phosphatidylserine-mediated immunosuppression, synergize with PD-1 blockade (96)
Combination regimens	Nintedanib + Pembrolizumab	Multi-target tyrosine kinases, PD-1	Inhibit fibroblast activation and angiogenesis, remodel TME, enhance ICI efficacy (97)

TME, tumor microenvironment; ICI, immune checkpoint inhibitor; TAMs, tumor-associated macrophages; VEGF, vascular endothelial growth factor.

#### 5.2.1 Small molecule inhibitors of inflammatory pathways

Small molecule inhibitors targeting core inflammatory pathways are currently the most widely studied direction. Among them, inhibitors targeting core inflammatory pathways such as NF-κB, JAK/STAT3, and MAPK have shown good anti-HCC effects in preclinical studies (84). For example, JAK inhibitors can significantly inhibit the proliferation and metastasis of HCC cells by blocking the IL-6/JAK/STAT3 signaling pathway, while improving the tumor immune microenvironment (85, 86). NF-κB inhibitors can effectively inhibit the inflammatory cascade in the liver, delaying the occurrence of liver fibrosis and HCC (41). The latest study by Mu confirmed that targeting the inflammatory pathway induced by ferroptosis can significantly inhibit HCC metastasis, while enhancing the therapeutic efficacy of sorafenib (37), providing a novel direction for the targeted therapy of HCC.

#### 5.2.2 Inflammation-targeted biological agents

Monoclonal antibodies targeting inflammatory cytokines are important biological agents for inflammation-targeted therapy. Among them, monoclonal antibodies against TNF-α, IL-6, and IL-1β have been widely used in autoimmune diseases, and clinical studies on their application in HCC treatment are currently ongoing (87). For example, tocilizumab, an anti-IL-6 receptor monoclonal antibody, can effectively block the IL-6/STAT3 signaling pathway, has shown good anti-HCC effects in preclinical studies, and can enhance the therapeutic response to immune checkpoint inhibitors (88). In addition, monoclonal antibodies targeting chemokines/chemokine receptors can inhibit the formation of the liver inflammatory microenvironment by blocking the recruitment of inflammatory cells, delaying the progression of HCC (89).

#### 5.2.3 Inflammation targeting combined with immunotherapy

Recent phase II trials have further verified the promising anti-tumor activity of inflammation-targeted agents combined with ICIs in advanced HCC, including stereotactic body radiotherapy combined with sintilimab plus bevacizumab, bavituximab plus pembrolizumab, and nintedanib plus pembrolizumab (90–97).

Key clinical challenges and evidence gaps: Despite promising preliminary data, several critical challenges remain for clinical translation. First, current evidence is mostly from single-arm phase II trials, with large-scale phase III randomized controlled trials confirming consistent overall survival benefits still lacking, which poses a key regulatory barrier to the formal clinical approval of these combination regimens. Second, balancing anti-tumor efficacy with treatment safety is a core clinical challenge: most HCC patients have underlying cirrhosis and impaired liver function, and the combination of anti-inflammatory agents with immune checkpoint inhibitors carries an elevated risk of both hematological toxicities and high-grade immune-related adverse events (irAEs), including immune-mediated hepatitis that can be life-threatening in patients with pre-existing liver dysfunction (72, 98). Third, there is an unmet need for predictive biomarkers to guide patient selection. The highly heterogeneous inflammatory landscape across HCC etiologies (as detailed in **Table 1**) leads to differential responses to inflammation-targeted therapy, yet current trials rarely stratify patients by etiology, and no validated biomarkers exist to identify beneficiaries of these combination regimens.

### 5.3 Inflammation-targeted primary prevention strategies for hepatocellular carcinoma

Primary prevention targeting the inflammatory “Wick” is the most cost-effective strategy to reduce HCC incidence, especially for high-risk populations with chronic liver disease. For patients with chronic viral hepatitis, effective antiviral therapy is the cornerstone of primary prevention, which can significantly inhibit viral replication, reduce hepatic inflammatory activity, delay fibrosis and cirrhosis progression, and ultimately lower HCC risk (93). For patients with MASH and ALD, lifestyle interventions including weight loss, alcohol abstinence, and metabolic risk factor control are the core strategies to alleviate chronic hepatic inflammation and reduce HCC risk (99).

In addition to etiology-specific intervention, anti-inflammatory agents have shown promising potential for HCC chemoprevention. Numerous epidemiological studies and clinical trials have confirmed that long-term use of non-steroidal anti-inflammatory drugs (NSAIDs), represented by aspirin, can significantly reduce HCC risk in patients with chronic liver disease by inhibiting the NF-κB and COX-2 inflammatory cascades (101). However, long-term NSAID use carries inherent safety risks, most notably an elevated incidence of gastrointestinal bleeding and ulceration. This risk is further amplified in cirrhotic patients, who often have coagulation disorders, portal hypertension, and gastroesophageal varices, making them more vulnerable to severe bleeding events. Therefore, NSAID use for HCC chemoprevention should be based on individualized risk-benefit assessment, with close monitoring of adverse events in high-risk cirrhotic populations.

## 6 Challenges and perspectives

Although a large number of research advances have been made in the mechanisms of inflammation-driven HCC occurrence and development, and inflammation-targeted diagnostic and therapeutic strategies have shown good application prospects, there are still many challenges in this field. First, inflammation has a well-documented bidirectional role in HCC, which is the core premise of inflammation-targeted intervention, but its regulatory mechanism and phenotypic transition node have not been fully elucidated. Acute resolving inflammation exerts a significant anti-tumor immune effect: it activates the anti-tumor function of innate immune cells (including M1-polarized macrophages, NK cells) and adaptive cytotoxic T lymphocytes, effectively clearing pre-malignant hepatocytes, inhibiting tumor initiation, and exerting a protective effect on the liver (102, 103). In contrast, chronic non-resolving inflammation, the core “Wick” of our proposed framework, exerts a sustained pro-tumor effect through the dual interwoven strands of inflammatory cells and cytokines: it drives hepatocyte genomic instability, activates hepatic stellate cells to promote liver fibrosis, and reshapes the immunosuppressive tumor microenvironment, ultimately promoting the whole process of HCC initiation and progression. Second, the inflammatory microenvironment of HCC is highly heterogeneous. There are significant differences in the cellular composition and molecular characteristics of the inflammatory microenvironment among HCC patients with different etiologies, different stages, and different genetic backgrounds. How to achieve precise targeted therapy based on the inflammatory characteristics of patients is a key bottleneck in clinical translation (97). In addition, most of the current inflammation-targeted therapeutic strategies are still in the preclinical research stage, and their safety and efficacy in clinical practice still need to be verified by large-sample clinical trials (68, 98). A further notable gap is the lack of sex-specific considerations: sex hormones significantly modulate hepatic inflammatory responses. For instance, estrogen has been shown to inhibit the IL-6/STAT3 signaling axis, providing a protective effect against HCC in females by dampening the second ‘strand’ of the inflammatory framework (104).

In the future, we will focus on the following concrete research pathways based on the “Single Wick, Dual Interwoven Strands” framework, combined with the multi-omics technologies mentioned above:

First, construct a spatiotemporal dynamic inflammatory atlas of HCC progression using single-cell sequencing and spatial transcriptomics, to precisely dissect the *in situ* interaction and dynamic change characteristics of inflammatory cells and cytokines (the dual interwoven strands) at different stages of HCC progression, and reveal novel regulatory mechanisms of inflammation-driven hepatocarcinogenesis (100).

Second, establish a molecular classification system of HCC based on inflammatory subtypes, through multi-omics profiling of the inflammatory microenvironment in large-scale HCC cohorts. This classification system can realize precise stratification of HCC patients, screen the beneficiary population of inflammation-targeted therapy, and effectively promote the clinical translation of related therapeutic strategies (101).

Third, develop individualized combination regimens guided by multi-omics inflammatory signatures: based on the inflammatory characteristics of patients identified by multi-omics technologies, optimize the combination regimen of inflammation targeting with immunotherapy, targeted therapy or local therapy, to break through the efficacy bottleneck of existing treatments and further improve the long-term survival prognosis of HCC patients (101).

In summary, chronic inflammation is the core driving force of HCC initiation and progression and exerts its pro-tumor effects via our proposed “Single Wick, Dual Interwoven Strands” framework. Chronic liver inflammation acts as the functional “Wick” that initiates and sustains the entire pro-tumor cascade, while the dynamic interweaving of inflammatory cells and inflammatory cytokines (the interactive regulatory network formed by the dual strands), rather than the simple sum of their independent effects, is the core driver that synergistically promotes the entire malignant course of HCC. The complexity of this interactive network has been precisely captured by advanced multi-omics technologies including single-cell sequencing and spatial transcriptomics mentioned in this review, which provide a solid technical basis for deciphering the spatiotemporal characteristics of the network. Moving forward, the core breakthrough direction in this field lies not only in dissecting the molecular mechanisms of inflammation-driven HCC, but more importantly in targeting this interwoven interactive regulatory network to develop precise and safe diagnostic and therapeutic strategies, which may lead to novel breakthroughs in the early prevention, precise treatment, and prognosis improvement of HCC.

## References

[1] Sung H Ferlay J Siegel RL Laversanne M Soerjomataram I Jemal A . Global cancer statistics 2020: GLOBOCAN estimates of incidence and mortality worldwide for 36 cancers in 185 countries. CA Cancer J Clin. (2021) 71:209–249. doi: 10.3322/caac.21660. PMID: 33538338

[2] Llovet JM Kelley RK Villanueva A Singal AG Pikarsky E Roayaie S . Hepatocellular carcinoma. Nat Rev Dis Primers. (2021) 7:6. doi: 10.1038/s41572-020-00240-3. PMID: 33479224 PMC13537433

[3] Hammerich L Tacke F . Hepatic inflammatory responses in liver fibrosis. Nat Rev Gastroenterol Hepatol. (2023) 20:633–646. doi: 10.1038/s41575-023-00807-x. PMID: 37400694

[4] Kisseleva T Ganguly S Murad R Wang A Brenner DA . Regulation of hepatic stellate cell phenotypes in metabolic dysfunction-associated steatohepatitis. Gastroenterology. (2025) 169:797–812. doi: 10.1053/j.gastro.2025.03.010. PMID: 40120772 PMC12416914

[5] Zhang Z Chen X Zhang B Zhang W Ding ZY . Fibrosis-driven hepatocarcinogenesis, metastasis and immune evasion: Mechanisms and therapeutic targets. Biochim Biophys Acta Rev Cancer. (2026) 1881:189517. doi: 10.1016/j.bbcan.2025.189517. PMID: 41421493

[6] Wang G Li J Bojmar L Chen H Li Z Tobias GC . Tumour extracellular vesicles and particles induce liver metabolic dysfunction. Nature. (2023) 618:374–382. doi: 10.1038/s41586-023-06114-4. PMID: 37225988 PMC10330936

[7] Jin K Shi Y Zhang H Zhangyuan G Wang F Li S . A TNFα/Miz1-positive feedback loop inhibits mitophagy in hepatocytes and propagates non-alcoholic steatohepatitis. J Hepatol. (2023) 79:403–416. doi: 10.1016/j.jhep.2023.03.039. PMID: 37040844

[8] Yahoo N Dudek M Knolle P Heikenwälder M . Role of immune responses in the development of NAFLD-associated liver cancer and prospects for therapeutic modulation. J Hepatol. (2023) 79:538–551. doi: 10.1016/j.jhep.2023.02.033. PMID: 36893854

[9] Zhang Y Jin D Zhu H Lin M Peng X . Hepatocytes and hepatic stellate cells carry different levels of DNA damage due to their sensitivity to oxidative stress in chronic hepatitis B. J Viral Hepat. (2025) 32:e70017. doi: 10.1111/jvh.70017. PMID: 39991880

[10] Kuchay MS Choudhary NS Ramos-Molina B . Pathophysiological underpinnings of metabolic dysfunction-associated steatotic liver disease. Am J Physiol Cell Physiol. (2025) 328:C1637–C1666. doi: 10.1152/ajpcell.00951.2024. PMID: 40244183

[11] Llovet JM Willoughby CE Singal AG Greten TF Heikenwälder M El-Serag HB . Nonalcoholic steatohepatitis-related hepatocellular carcinoma: pathogenesis and treatment. Nat Rev Gastroenterol Hepatol. (2023) 20:487–503. doi: 10.1038/s41575-023-00754-7. PMID: 36932227 PMC12165718

[12] Narmada BC Khakpoor A Shirgaonkar N Narayanan S Aw PPK Singh M . Single-cell landscape of functionally cured chronic hepatitis B patients reveals activation of innate and altered CD4-CTL-driven adaptive immunity. J Hepatol. (2024) 81:42–61. doi: 10.1016/j.jhep.2024.02.012. PMID: 38423478

[13] Guerra-Veloz MF Shah S Emmanouil B Olsen M George R Selemani S . Setting the record straight: Utility and outcomes in patients with HCV related HCC. J Viral Hepat. (2026) 33:e70148. doi: 10.1111/jvh.70148. PMID: 41665509

[14] Hazari Y Chevet E Bailly-Maitre B Hetz C . ER stress signaling at the interphase between MASH and HCC. Hepatology. (2026) 83:387–408. doi: 10.1097/HEP.0000000000000893. PMID: 38626349

[15] Alvarado-Tapias E Pose E Gratacós-Ginès J Clemente-Sánchez A López-Pelayo H Bataller R . Alcohol-associated liver disease: Natural history, management and novel targeted therapies. Clin Mol Hepatol. (2025) 31:S112–S133. doi: 10.3350/cmh.2024.0709. PMID: 39481875 PMC11925442

[16] Jiang Z Wu Y Miao Y Deng K Yang F Xu S . HCCDB v2.0: Decompose expression variations by single-cell RNA-seq and spatial transcriptomics in HCC. Genomics Proteomics Bioinf. (2024) 22:qzae011. doi: 10.1093/gpbjnl/qzae011. PMID: 38886186 PMC11423853

[17] Huang HY Chen YZ Zhao C Zheng XN Yu K Yue JX . Alternations in inflammatory macrophage niche drive phenotypic and functional plasticity of Kupffer cells. Nat Commun. (2024) 15:9337. doi: 10.1038/s41467-024-53659-7. PMID: 39472435 PMC11522483

[18] Yan Y Bai S Han H Dai J Niu L Wang H . Knockdown of trem2 promotes proinflammatory microglia and inhibits glioma progression via the JAK2/STAT3 and NF-κB pathways. Cell Commun Signal. (2024) 22:272. doi: 10.1186/s12964-024-01642-6. PMID: 38750472 PMC11094905

[19] Ma H Cao T Zhang F Sun D Chen L Lin Y . Nuclear Pirin promotes HCC by acting as a key inflammation-facilitating factor. Gut. (2025) 75:1016–29. doi: 10.1136/gutjnl-2024-334087. PMID: . Epub ahead of print. 40579121 PMC13151453

[20] Liu F Li X Zhang Y Ge S Shi Z Liu Q . Targeting tumor-associated macrophages to overcome immune checkpoint inhibitor resistance in hepatocellular carcinoma. J Exp Clin Cancer Res. (2025) 44:227. doi: 10.1186/s13046-025-03490-9. PMID: 40764998 PMC12323087

[21] Patseas D El-Masry A Liu Z Ramachandran P Triantafyllou E . Myeloid cells in chronic liver inflammation. Cell Mol Immunol. (2025) 22:1237–1261. doi: 10.1038/s41423-025-01324-1. PMID: 40721870 PMC12479923

[22] Deng Z Mei S Ouyang Z Wang R Wang L Zou B . Dysregulation of gut microbiota stimulates NETs-driven HCC intrahepatic metastasis: therapeutic implications of healthy faecal microbiota transplantation. Gut Microbes. (2025) 17:2476561. doi: 10.1080/19490976.2025.2476561. PMID: 40099491 PMC11925110

[23] Tilg H Adolph TE Tacke F . Therapeutic modulation of the liver immune microenvironment. Hepatology. (2023) 78:1581–1601. doi: 10.1097/HEP.0000000000000386. PMID: 37057876

[24] Park H Lee S Lee J Moon H Ro SW . Exploring the JAK/STAT signaling pathway in hepatocellular carcinoma: Unraveling signaling complexity and therapeutic implications. Int J Mol Sci. (2023) 24:13764. doi: 10.3390/ijms241813764. PMID: 37762066 PMC10531214

[25] Weinberg SE Chandel NS . Mitochondria reactive oxygen species signaling-dependent immune responses in macrophages and T cells. Immunity. (2025) 58:1904–1921. doi: 10.1016/j.immuni.2025.07.012. PMID: 40763730 PMC12371701

[26] Yang LY Luo Q Lu L Zhu WW Sun HT Wei R . Increased neutrophil extracellular traps promote metastasis potential of hepatocellular carcinoma via provoking tumorous inflammatory response. J Hematol Oncol. (2020) 13:3. doi: 10.1186/s13045-019-0836-0. PMID: 31907001 PMC6945602

[27] Hong YM Yoon KT Hwang TH Cho M . Pretreatment peripheral neutrophils, lymphocytes and monocytes predict long-term survival in hepatocellular carcinoma. BMC Cancer. (2020) 20:937. doi: 10.1186/s12885-020-07105-8. PMID: 32993594 PMC7526162

[28] Curio S Belz GT . The unique role of innate lymphoid cells in cancer and the hepatic microenvironment. Cell Mol Immunol. (2022) 19:1012–1029. doi: 10.1038/s41423-022-00901-1. PMID: 35962192 PMC9424527

[29] Ajith A Merimi M Arki MK Hossein-Khannazer N Najar M Vosough M . Immune regulation and therapeutic application of T regulatory cells in liver diseases. Front Immunol. (2024) 15:1371089. doi: 10.3389/fimmu.2024.1371089. PMID: 38571964 PMC10987744

[30] He Y Luo J Zhang G Jin Y Wang N Lu J . Single-cell profiling of human CD127+ innate lymphoid cells reveals diverse immune phenotypes in hepatocellular carcinoma. Hepatology. (2022) 76:1013–1029. doi: 10.1002/hep.32444. PMID: 35243668 PMC9790738

[31] Ayers M Lunceford J Nebozhyn M Murphy E Loboda A Kaufman DR . IFN-γ-related mRNA profile predicts clinical response to PD-1 blockade. J Clin Invest. (2017) 127:2930–2940. doi: 10.1172/JCI91190. PMID: 28650338 PMC5531419

[32] Wang H Tsung A Mishra L Huang H . Regulatory T cell: a double-edged sword from metabolic-dysfunction-associated steatohepatitis to hepatocellular carcinoma. EBioMedicine. (2024) 101:105031. doi: 10.1016/j.ebiom.2024.105031. PMID: 38401419 PMC10904199

[33] Ruf B Bruhns M Babaei S Kedei N Ma L Revsine M . Tumor-associated macrophages trigger MAIT cell dysfunction at the HCC invasive margin. Cell. (2023) 186:3686–3705.e32. doi: 10.1016/j.cell.2023.07.023. PMID: 37595566 PMC10461130

[34] Lurje I Hammerich L Tacke F . Dendritic cell and T cell crosstalk in liver fibrogenesis and hepatocarcinogenesis: Implications for prevention and therapy of liver cancer. Int J Mol Sci. (2020) 21:7378. doi: 10.3390/ijms21197378. PMID: 33036244 PMC7583774

[35] Xu Q Chen J Liu Y Yu J Zhao F Shu Q . Exosomal crosstalk: the metastatic language of hepatocellular carcinoma. Front Immunol. (2026) 16:1701305. doi: 10.3389/fimmu.2025.1701305. PMID: 41567219 PMC12815768

[36] Rico Montanari N Anugwom CM Boonstra A Debes JD . The role of cytokines in the different stages of hepatocellular carcinoma. Cancers. (2021) 13:4876. doi: 10.3390/cancers13194876. PMID: 34638361 PMC8508513

[37] Mu M Huang CX Qu C Li PL Wu XN Yao W . Targeting ferroptosis-elicited inflammation suppresses hepatocellular carcinoma metastasis and enhances sorafenib efficacy. Cancer Res. (2024) 84:841–854. doi: 10.1158/0008-5472.CAN-23-1796. PMID: 38231484

[38] Rosenberg N Van Haele M Lanton T Brashi N Bromberg Z Adler H . Combined hepatocellular-cholangiocarcinoma derives from liver progenitor cells and depends on senescence and IL-6 trans-signaling. J Hepatol. (2022) 77:1631–1641. doi: 10.1016/j.jhep.2022.07.029. PMID: 35988690

[39] Yan L Xu F Dai CL . Relationship between epithelial-to-mesenchymal transition and the inflammatory microenvironment of hepatocellular carcinoma. J Exp Clin Cancer Res. (2018) 37:203. doi: 10.1186/s13046-018-0887-z. PMID: 30157906 PMC6114477

[40] Bi W Li X Jiang Y Gao T Zhao H Han Q . Tumor-derived exosomes induce neutrophil infiltration and reprogramming to promote T-cell exhaustion in hepatocellular carcinoma. Theranostics. (2025) 15:2852–2869. doi: 10.7150/thno.104557. PMID: 40083930 PMC11898284

[41] González-García K Zertuche-Martínez C Reyes-Avendaño I Reyes-Jiménez E Muriel P Villa-Treviño S . An integrated systematic review and meta-analysis from the bloodstream to identify potential biomarkers for ALD, MASLD, and HCC without a viral background. J Gastroenterol Hepatol. (2026) 41:502–515. doi: 10.1111/jgh.70228. PMID: 41481046

[42] Karin M . Nuclear factor-kappaB in cancer development and progression. Nature. (2006) 441:431–436. doi: 10.1038/nature04870. PMID: 16724054

[43] Liu T Zhang L Joo D Sun SC . NF-κB signaling in inflammation. Signal Transduct Target Ther. (2017) 2:17023. doi: 10.1038/sigtrans.2017.23. PMID: 29158945 PMC5661633

[44] Verboom L Martens A Priem D Hoste E Sze M Vikkula H . OTULIN prevents liver inflammation and hepatocellular carcinoma by inhibiting FADD- and RIPK1 kinase-mediated hepatocyte apoptosis. Cell Rep. (2020) 30:2237–2247.e6. doi: 10.1016/j.celrep.2020.01.024. PMID: 32075762

[45] Ishteyaque S Singh G Yadav KS Verma S Sharma RK Sen S . Cooperative STAT3-NFkB signaling modulates mitochondrial dysfunction and metabolic profiling in hepatocellular carcinoma. Metabolism. (2024) 152:155771. doi: 10.1016/j.metabol.2023.155771. PMID: 38184165

[46] Sun H Yang W Tian Y Zeng X Zhou J Mok MTS . An inflammatory-CCRK circuitry drives mTORC1-dependent metabolic and immunosuppressive reprogramming in obesity-associated hepatocellular carcinoma. Nat Commun. (2018) 9:5214. doi: 10.1038/s41467-018-07402-8. PMID: 30523261 PMC6283830

[47] Zhang C Zhou Y Hu M Pan Y Chen X Sun Q . PLOD1 promotes the malignancy of hepatocellular carcinoma by facilitating the NF-κB/IL-6/STAT3-dependent TCA cycle. JHEP Rep. (2025) 7:101329. doi: 10.1016/j.jhepr.2025.101329. PMID: 40290518 PMC12023786

[48] Li J Huang Y Li J Shi M Xiao Y Du F . Metabolic reprogramming-driven resistance to multi-kinase inhibitors in hepatocellular carcinoma: molecular mechanisms and therapeutic opportunities. Mol Cancer. (2026) 25:37. doi: 10.1186/s12943-026-02578-w. PMID: 41593671 PMC12918548

[49] Mohammed S Thadathil N Selvarani R Nicklas EH Wang D Miller BF . Necroptosis contributes to chronic inflammation and fibrosis in aging liver. Aging Cell. (2021) 20:e13512. doi: 10.1111/acel.13512. PMID: 34761505 PMC8672775

[50] Huang QL Feng DY Zhao WX Zhang ZY Jiang ZH Han MZ . A unique intercellular feedforward loop from HK1 to TGF-β1 promotes the progression of hepatocellular carcinoma. J Extracell Vesicles. (2026) 15:e70255. doi: 10.1002/jev2.70255. PMID: 41806338 PMC12974909

[51] Wu R Wu R Kong X Wang X Duan Y Cao S . Neutrophil-macrophage crosstalk via NETs-IL-17/VEGF/S100A9 axis promotes hepatocellular carcinoma progression. J Exp Clin Cancer Res. (2025) 45:27. doi: 10.1186/s13046-025-03618-x. PMID: 41469919 PMC12853889

[52] Chen C Wang Z Ding Y Qin Y . Tumor microenvironment-mediated immune evasion in hepatocellular carcinoma. Front Immunol. (2023) 14:1133308. doi: 10.3389/fimmu.2023.1133308. PMID: 36845131 PMC9950271

[53] Tang T Li Y Xiyun N Wu H Fan L Zhang X . Crosstalk between SPP1+ macrophages and ITGA5+ fibroblasts promotes hepatocellular carcinoma metastasis. Hepatol Commun. (2026) 10:e00907. doi: 10.1097/HC9.0000000000000907. PMID: 41758046 PMC12948000

[54] Zhang W Zhangyuan G Wang F Jin K Shen H Zhang L . The zinc finger protein Miz1 suppresses liver tumorigenesis by restricting hepatocyte-driven macrophage activation and inflammation. Immunity. (2021) 54:1168–1185.e8. doi: 10.1016/j.immuni.2021.04.027. PMID: 34038747

[55] Lin L Chen S Wang H Gao B Kallakury B Bhuvaneshwar K . SPTBN1 inhibits inflammatory responses and hepatocarcinogenesis via the stabilization of SOCS1 and downregulation of p65 in hepatocellular carcinoma. Theranostics. (2021) 11:4232–4250. doi: 10.7150/thno.49819. PMID: 33754058 PMC7977457

[56] Casari M Siegl D Deppermann C Schuppan D . Macrophages and platelets in liver fibrosis and hepatocellular carcinoma. Front Immunol. (2023) 14:1277808. doi: 10.3389/fimmu.2023.1277808. PMID: 38116017 PMC10728659

[57] Hanahan D . Hallmarks of cancer: New dimensions. Cancer Discov. (2022) 12:31–46. doi: 10.1158/2159-8290.CD-21-1059. PMID: 35022204

[58] Sengez B Carr BI Alotaibi H . EMT and inflammation: Crossroads in HCC. J Gastrointest Cancer. (2023) 54:204–212. doi: 10.1007/s12029-021-00801-z. PMID: 35020133

[59] Lamouille S Xu J Derynck R . Molecular mechanisms of epithelial-mesenchymal transition. Nat Rev Mol Cell Biol. (2014) 15:178–196. doi: 10.1038/nrm3758. PMID: 24556840 PMC4240281

[60] Derynck R Weinberg RA . EMT and cancer: More than meets the eye. Dev Cell. (2019) 50:742–745. doi: 10.1016/j.devcel.2019.08.010. PMID: 31063750 PMC7672963

[61] Potente M Gerhardt H Carmeliet P . Basic and therapeutic aspects of angiogenesis. Cell. (2011) 146:873–887. doi: 10.1016/j.cell.2011.08.039. PMID: 21925313

[62] Gui M Huang S Li S Chen Y Cheng F Liu Y . Integrative single-cell transcriptomic analyses reveal the cellular ontological and functional heterogeneities of primary and metastatic liver tumors. J Transl Med. (2024) 22:206. doi: 10.1186/s12967-024-04947-9. PMID: 38414027 PMC10898050

[63] Apte RS Chen DS Ferrara N . VEGF in signaling and disease: Beyond discovery and development. Cell. (2019) 176:1248–1264. doi: 10.1016/j.cell.2019.01.021. PMID: 30849371 PMC6410740

[64] Chen DS Mellman I . Elements of cancer immunity and the cancer-immune set point. Nature. (2017) 541:321–330. doi: 10.1038/nature21349. PMID: 28102259

[65] Ribas A Wolchok JD . Cancer immunotherapy using checkpoint blockade. Science. (2018) 359:1350–1355. doi: 10.1126/science.aar4060. PMID: 29567705 PMC7391259

[66] Zhang T Zhang P Zhang H Zhang Z Ran J . Exploring targeted delivery systems for boron neutron capture therapy and its potential as a promising therapeutic modality for hepatocellular carcinoma. Cancer Biother Radiopharm. (2026) 41:6–14. doi: 10.1177/10849785251370873. PMID: 40854589

[67] Zhang S Yuan L Danilova L Mo G Zhu Q Deshpande A . Spatial transcriptomics analysis of neoadjuvant cabozantinib and nivolumab in advanced hepatocellular carcinoma identifies independent mechanisms of resistance and recurrence. Genome Med. (2023) 15:72. doi: 10.1186/s13073-023-01218-y. PMID: 37723590 PMC10506285

[68] Zhou L Zhang W Liu Z Xie Y Jiang K . Immune cell metabolic reprogramming in hepatocellular carcinoma: mechanisms, tumor microenvironment, and future immunotherapeutic directions. Front Immunol. (2026) 16:1697675. doi: 10.3389/fimmu.2025.1697675, PMID: 41601626 PMC12833076

[69] Ju Y Xu K Chen X Wu T Yuan Y . Metabolic-immune microenvironment crosstalk mediating ICI resistance in MASH-HCC. Trends Endocrinol Metab. (2026) 37:262–276. doi: 10.1016/j.tem.2025.11.006. PMID: 40695685

[70] Pavlova NN Thompson CB . The emerging hallmarks of cancer metabolism. Cell Metab. (2016) 23:27–47. doi: 10.1016/j.cmet.2015.12.006. PMID: 26771115 PMC4715268

[71] Liberti MV Locasale JW . The Warburg effect: How does it benefit cancer cells?. Trends Biochem Sci. (2016) 41:211–218. doi: 10.1016/j.tibs.2015.12.001. PMID: 26778478 PMC4783224

[72] Zhang Y Xiong S Zheng W Liu J Zhao Y Lv J . Reshaping the immunosuppressive niche in hepatocellular carcinoma: crosstalk networks, metabolic reprogramming, and therapeutic strategies. Biochem Pharmacol. (2026) 248:117858. doi: 10.1016/j.bcp.2026.117858. PMID: 41794264

[73] Dong H Shao M Tao Z Wang Z Gao Z Qiu W . Targeting inflammation in hepatocellular carcinoma: emerging nanotherapeutic strategies for remodeling immunosuppressive microenvironments. Biomater Sci. (2026) 14:1162–1179. doi: 10.1039/d5bm01798j. PMID: 41631443

[74] Ramaite FT Nkadimeng SM . Targeting inflammatory pathways in hepatocellular carcinoma: recent developments. Discov Oncol. (2025) 16:1174. doi: 10.1007/s12672-025-03035-8. PMID: 40544399 PMC12183140

[75] Banerjee A Farci P . Fibrosis and hepatocarcinogenesis: Role of gene-environment interactions in liver disease progression. Int J Mol Sci. (2024) 25:8641. doi: 10.3390/ijms25168641. PMID: 39201329 PMC11354981

[76] Zhou Z Xu X . Extracellular matrix stiffness in hepatocellular carcinoma: mechanisms and targeted therapeutic strategies. Front Immunol. (2026) 17:1773909. doi: 10.3389/fimmu.2026.1773909. PMID: 41890723 PMC13014048

[77] Jiang Z Wang H Li H Chen Z Sun B . Single-cell profiling reveals lineage-specific fibroblast stromal subtypes drive ECM remodeling and immune modulation in the hepatocellular carcinoma tumor microenvironment. Med Oncol. (2026) 43:108. doi: 10.1007/s12032-025-03220-3. PMID: 41483290 PMC12764699

[78] Dutta P Kumar V Sharma A . Systemic inflammation-based prognostic scores in hepatocellular carcinoma: An update. Eur J Surg Oncol. (2020) 46:1594–1603. doi: 10.1016/j.ejso.2020.04.020. PMID: 32418755

[79] Rocco A Sgamato C Pelizzaro F Simeon V Coccoli P Compare D . Systemic inflammatory response markers improve the discrimination for prognostic model in hepatocellular carcinoma. Hepatol Int. (2025) 19:915–928. doi: 10.1007/s12072-025-10806-9. PMID: 40131621 PMC12287231

[80] Laschtowitz A Lambrecht J Puengel T Tacke F Mohr R . Serum CXCL5 detects early hepatocellular carcinoma and indicates tumor progression. Int J Mol Sci. (2023) 24:5295. doi: 10.3390/ijms24065295. PMID: 36982370 PMC10049661

[81] Shi W Jin W Hong L Wu H Hu S . Clinical value of blood routine and tumor markers in differentiating hepatocellular carcinoma from intrahepatic cholangiocarcinoma. Medicine. (2025) 104:e41899. doi: 10.1097/MD.0000000000041899. PMID: 40128070 PMC11936544

[82] Li S Zeng Q Liang R Long J Liu Y Xiao H . Using systemic inflammatory markers to predict microvascular invasion before surgery in patients with hepatocellular carcinoma. Front Surg. (2022) 9:833779. doi: 10.3389/fsurg.2022.833779. PMID: 35310437 PMC8931769

[83] Utsumi M Inagaki M Kitada K Tokunaga N Yunoki K Sakurai Y . Predictive values of sarcopenia and systemic inflammation-based markers in advanced hepatocellular carcinoma after hepatectomy. Asian J Surg. (2024) 47:3039–3047. doi: 10.1016/j.asjsur.2024.02.004. PMID: 38388270

[84] He G Karin M . NF-κB and STAT3 - key players in liver inflammation and cancer. Cell Res. (2011) 21:159–168. doi: 10.1038/cr.2010.176. PMID: 21187858 PMC3193410

[85] Chen J Ji T Zhao J Li G Zhang J Jin R . Sorafenib-resistant hepatocellular carcinoma stratified by phosphorylated ERK activates PD-1 immune checkpoint. Oncotarget. (2016) 7:41274–41284. doi: 10.18632/oncotarget.8978. PMID: 27129180 PMC5173058

[86] Li L Cheng J Guo HL Liu Q Wang N Wu CX . Augmenter of liver regeneration enhances hepatocellular carcinoma cell growth through COX-2-associated signaling. Cancer Genomics Proteomics. (2026) 23:109–126. doi: 10.21873/cgp.20564. PMID: 41482353 PMC12758719

[87] Balkwill F . TNF-α in promotion and progression of cancer. Cancer Metastasis Rev. (2006) 25:409–416. doi: 10.1007/s10555-006-9028-9. PMID: 16951987

[88] Geng X Li J Wu B Wang W Li Z Liu S . SLC25A39 facilitates sorafenib resistance in hepatocellular carcinoma by inhibiting mitochondrial oxidative stress-induced ferroptosis. Cancer Cell Int. (2026) 26:63. doi: 10.1186/s12935-025-04151-9. PMID: 41495816 PMC12870238

[89] Hughes CE Nibbs RJB . A guide to chemokines and their receptors. FEBS J. (2018) 285:2944–2971. doi: 10.1111/febs.14468. PMID: 29637711 PMC6120486

[90] Chan SL Ryoo BY Mo F Chan LL Cheon J Li L . Multicentre phase II trial of cabozantinib in patients with hepatocellular carcinoma after immune checkpoint inhibitor treatment. J Hepatol. (2024) 81:258–264. doi: 10.1016/j.jhep.2024.03.033. PMID: 38570034

[91] Zhou Z Chen J Lu N Wu J Li Y Shen Y . Matrix stiffening-driven hepatocellular carcinoma progression through OASL-mediated cGAS-STING repression and subsequent macrophage activation. Int Immunopharmacol. (2026) 168:115956. doi: 10.1016/j.intimp.2025.115956. PMID: 41330170

[92] Briukhovetska D Dörr J Endres S Libby P Dinarello CA Kobold S . Interleukins in cancer: from biology to therapy. Nat Rev Cancer. (2021) 21:481–499. doi: 10.1038/s41568-021-00363-z. PMID: 34083781 PMC8173513

[93] Pyonteck SM Akkari L Schuhmacher AJ Bowman RL Sevenich L Quail DF . CSF1R inhibition alters macrophage polarization and blocks glioma progression. Nat Med. (2013) 19:1264–1272. doi: 10.1038/nm.3337. PMID: 24056773 PMC3840724

[94] Tang J Yang Y Liu D Wang B Lin Z Wu Z . Stereotactic body radiotherapy with sintilimab and bevacizumab biosimilar in anti-PD-1 refractory hepatocellular carcinoma: the ReUNION-1 phase 2 trial. Nat Commun. (2025) 17:823. doi: 10.1038/s41467-025-67528-4. PMID: 41413036 PMC12823611

[95] Abdelrahim M Esmail A Al-Judaibi B . Durvalumab and bevacizumab plus transarterial chemoembolization in unresectable hepatocellular carcinoma: a new paradigm?. Transl Gastroenterol Hepatol. (2026) 11:1. doi: 10.21037/tgh-25-34. PMID: 41675315 PMC12887335

[96] Hsiehchen D Beg MS Kainthla R Lohrey J Kazmi SM Khosama L . The phosphatidylserine targeting antibody bavituximab plus pembrolizumab in unresectable hepatocellular carcinoma: a phase 2 trial. Nat Commun. (2024) 15:2178. doi: 10.1038/s41467-024-46542-y. PMID: 38467639 PMC10928173

[97] Baldini C Danlos FX Varga A Texier M Halse H Mouraud S . Safety, recommended dose, efficacy and immune correlates for nintedanib in combination with pembrolizumab in patients with advanced cancers. J Exp Clin Cancer Res. (2022) 41:217. doi: 10.1186/s13046-022-02423-7. PMID: 35794623 PMC9260998

[98] Daetwyler E Wallrabenstein T König D Cappelli LC Naidoo J Zippelius A . Corticosteroid-resistant immune-related adverse events: a systematic review. J Immunother Cancer. (2024) 12:e007409. doi: 10.1136/jitc-2023-007409. PMID: 38233099 PMC10806650

[99] Polyzos SA Chrysavgis L Vachliotis ID Chartampilas E Cholongitas E . Nonalcoholic fatty liver disease and hepatocellular carcinoma: Insights in epidemiology, pathogenesis, imaging, prevention and therapy. Semin Cancer Biol. (2023) 93:20–35. doi: 10.1016/j.semcancer.2023.04.010. PMID: 37149203

[100] Terrault NA Lok ASF McMahon BJ Chang KM Hwang JP Jonas MM . Update on prevention, diagnosis, and treatment of chronic hepatitis B: AASLD 2018 hepatitis B guidance. Hepatology. (2018) 67:1560–1599. doi: 10.1002/hep.29800. PMID: 29405329 PMC5975958

[101] Simon TG Duberg AS Aleman S Chung RT Chan AT Ludvigsson JF . Association of aspirin with hepatocellular carcinoma and liver-related mortality. N Engl J Med. (2020) 382:1018–1028. doi: 10.1056/NEJMoa1912035. PMID: 32160663 PMC7317648

[102] Grivennikov SI Greten FR Karin M . Immunity, inflammation, and cancer. Cell. (2010) 140:883–899. doi: 10.1016/j.cell.2010.01.025. PMID: 20303878 PMC2866629

[103] Geh D Leslie J Rumney R Reeves HL Bird TG Mann DA . Neutrophils as potential therapeutic targets in hepatocellular carcinoma. Nat Rev Gastroenterol Hepatol. (2022) 19:257–273. doi: 10.1038/s41575-021-00568-5. PMID: 35022608

[104] Zhou P Sun F Lin P Yan Y Liu J Zhou Y . Estrogen inhibits hepatocellular carcinoma progression dependent on HOXA11-AS/HOXA11. Transl Oncol. (2025) 57:102404. doi: 10.1016/j.tranon.2025.102404. PMID: 40344916 PMC12138468

